# Retraction: A mathematical model explains saturating axon guidance responses to molecular gradients

**DOI:** 10.7554/eLife.37048

**Published:** 2018-04-12

**Authors:** Huyen Nguyen, Peter Dayan, Zac Pujic, Justin Cooper-White, Geoffrey J Goodhill

Nguyen H, Dayan P, Pujic Z, Cooper-White J, Goodhill GJ. 2016. A mathematical model explains saturating axon guidance responses to molecular gradients. *eLife* **5**:e12248. doi: 10.7554/eLife.12248.

Published 2, February 2016

We are retracting the *eLife* paper cited above. In attempting to reproduce the quantitative analysis of the experimental axon trajectory data we encountered a number of errors and inconsistences. The first author (HN) could not supply the source data for the control experiments contributing to Figures 4, 5, 6, 8, and 11, or the final version of the code that generated these figures. We found that the Excel spreadsheets supplied with the figures were incorrect, as were the boxes drawn in Figures 9 and 10. Although we found no problem with the mathematical model or theoretical analysis, Figures 12 and 13 rely on parameters obtained from fitting data that is no longer available for verification.

We hope to be able to address these issues with new experimental data in the future. However, the changes that would be required to correct the paper are sufficiently extensive that its overall conclusions must currently be considered to be in doubt. We are therefore in agreement that retraction is the appropriate course of action at this time. We sincerely apologise.

